# Efficacy and safety of Xinkeshu in the treatment of angina pectoris of coronary heart disease: A protocol for systematic review and meta-analysis

**DOI:** 10.1097/MD.0000000000027407

**Published:** 2021-10-08

**Authors:** Maoxia Fan, Dong Guo, Yiming Wang, Yongcheng Liu, Jisen Zhao, Zhou Yu

**Affiliations:** a The First Clinical Medical College of Shandong University of Traditional Chinese Medicine, Shandong Province, China; b Teacher Development Center of Shandong University of Traditional Chinese Medicine, Shandong Province, China.

**Keywords:** angina pectoris of coronary heart disease, protocol, systematic review, Xinkeshu

## Abstract

**Background:**

The incidence of angina pectoris (AP) of coronary heart disease (CHD) is increasing in the world, which seriously affects people's lives and brings a huge economic burden. The clinical research on Xinkeshu (XKS) in the treatment of AP of CHD has been increasing. However, there is no systematic review and meta-analysis. This study intends to provide a basis for systematically evaluating the efficacy and safety of XKS combined with conventional western medicine in the treatment of AP of CHD.

**Methods:**

CNKI, Wanfang, VIP, Web of Science, PubMed, Cochrane Library, and EMbase databases were searched for the period from the establishment of the database to August 31, 2021. The clinical randomized controlled trials of XKS in the treatment of AP of CHD were collected. Two systematic reviewers independently selected the literature, extracted the data, and evaluated the quality according to the inclusion and exclusion criteria. The methodological quality of the literature was evaluated using Cochrane Handbook 5.3.0 bias risk assessment tool, RevMan 5.3.0 software was used for meta-analysis and GRADE3.6 evidence quality grading system was used to evaluate the quality.

**Results:**

This study intended to evaluate the efficacy and safety of XKS in the treatment of AP of CHD from many aspects, including the frequency of AP, the duration of AP, the dosage of nitroglycerin, and the efficacy of ECG (total effective rate = markedly effective + effective). The secondary indicators included the efficacy of AP (total effective rate = significant + effective), blood lipids (triglyceride, total cholesterol, low-density lipoprotein cholesterol, and high-density lipoprotein cholesterol), hemorheology (whole blood viscosity, plasma viscosity, and fibrinogen), serum factors (C-reactive protein, endothelin-1, homocysteine, and nitric oxide), and adverse drug reactions.

**Conclusion:**

The conclusion of the systematic review intended to provide clear evidence of clinical application of XKS combined with conventional western medicine in the treatment of AP of CHD, which can be widely used in the clinic.

## 1 Introduction

Coronary heart disease (CHD) mainly refers to heart disease caused by the decrease of cardiac blood supply and oxygen supply caused by coronary artery stenosis or occlusion.^[1]^ In addition, angina pectoris (AP) is defined as chest pain or cardiac origin discomfort caused by a temporary imbalance between myocardial supply and demand.^[2]^ The main risk factors include hypertension, diabetes, dyslipidemia, smoking, etc. The management of AP of CHD is very important. The total number of deaths from CHD in China in 2013 was 1.394 million, with an increase of 90% from 1990 according to a research report by the Chinese Center for Disease Control and Prevention. The results of a number of studies show that the incidence and death toll of CHD in China will continue to increase with the intensification of the aging process.^[3]^ However, the available anti-ischemic drugs are limited due to the limitations of its anti-hemodynamic and electrophysiological effects.^[4,5]^ It has been reported that percutaneous revascularization can relieve symptoms better than anti- AP drugs, but this advantage will diminish with the passage of time.^[6]^ Traditional Chinese medicine (TCM), which has a long history in China and other Asian countries, can provide more options for CHD and AP. In China, most patients with AP of CHD also seek the help of Chinese herbal medicine in addition to routine treatment.^[7]^ In addition, clinical trials have shown that ingredients derived from Chinese herbal medicine are beneficial to patients with AP of CHD.^[8]^

AP of CHD belongs to “chest arthralgia,” “heartache.” The main pathogenesis of AP of CHD is “heart pulse stasis,” “qi stasis” according to traditional Chinese medicine. In addition, “activating qi and dredging collaterals, activating blood circulation and removing blood stasis” is its main treatment principle. Clinical observation confirmed that the combined application of traditional Chinese medicine on the basis of conventional drug treatment can improve the clinical therapeutic effect of patients with AP of CHD to a certain extent.^[9]^ At present, Xinkeshu (XKS) combined with conventional western medicine in the treatment of AP of CHD has been paid more and more attention and widely used. In addition, the results of clinical studies have been published continuously. However, clinical randomized controlled trials (RCT) with small samples are lack of systematic evaluation, and their use lacks the support of clinical evidence because all clinical studies are carried out in a single center. The purpose of this study is to provide a protocol for the systematic evaluation of the clinical RCT study of XKS in the treatment of AP of CHD. In addition, it intends to evaluate its clinical efficacy and safety, providing a reference for clinical practice and decision-making.

## 2 Methods

### 2.1 Inclusion criteria

#### 2.1.1 Type

RCT of AP of CHD treated by XKS combined with conventional western medicine.

#### 2.1.2 Objects

The diagnostic criteria of AP of CHD can be met according to the diagnostic criteria of syndrome elements of AP of CHD. In addition, there are no restrictions on age, sex, history of tobacco and alcohol, and so on.

#### 2.1.3 Intervention measures

The control group was only treated with the same routine western medicine. In clinical, the main drugs included anticoagulation, anti-platelet aggregation, β-receptor blockers, nitrates, angiotensin-converting enzyme inhibitors, statins regulating blood lipids, and other drugs. At the same time, the treatment group was treated with XKS capsule or XKS tablet or XKS decoction combined with conventional western medicine.

#### 2.1.4 Outcome index

The criteria for judging the expected outcome were clear, including the frequency of AP, the duration of AP, the dosage of nitroglycerin, and the curative effect of ECG (total effective rate = markedly effective + effective). The secondary indicators were the efficacy of AP (total effective rate = significant + effective), blood lipids (triglyceride, total cholesterol, low-density lipoprotein cholesterol, and high-density lipoprotein cholesterol), hemorheology (whole blood viscosity, plasma viscosity, and fibrinogen), serum factors (C-reactive protein, endothelin-1, homocysteine, and nitric oxide), and adverse drug reactions.

### 2.2 Exclusion criteria

The treatment group was only treated with XKS capsule or XKS tablet or XKS decoction in the non-clinical RCT. Besides, the intervention measures did not meet the inclusion criteria, and the diagnosis was not clear. Besides, the course of treatment was not clear. In addition to the adverse reactions, it does not include any of the outcome indexes. Moreover, review and animal experiments and other literature were excluded.

### 2.3 Electronic retrieval

China National knowledge Infrastructure, Wanfang, VIP, Web of Science, PubMed, Cochrane Library, and EMbase databases were searched for the period from the establishment of the database to August 31, 2021. References that meet the inclusion criteria of the study are intended to be reviewed 1 by 1 to avoid omissions. XKS and AP of CHD and their synonyms were set as search words. Taking the retrieval strategy in PubMed as an example (Table 1).

**Table 1:** Search strategy for PubMed.

Number	Search terms
1	#1 Search (“Angina Pectoris of Coronary Heart Disease”[Title/Abstract]) OR (“Coronary Heart Disease Angina Pectoris”[Title/Abstract]) OR (“Coronary Diseases Angina Pectoris”[Title/Abstract]) OR (“Angina Pectoris”[MeSH Terms]) OR (“Angina, Stable”[Title/Abstract]) OR (“Angina, Unstable”[Title/Abstract]) OR (“Angina Pectoris, Variant”[Title/Abstract]) OR (“Microvascular Angina”[Title/Abstract])
2	#2 Search (“Xinkeshu Capsules”) OR (“Xinkeshu Tablets”) OR (“Xinkeshu Decoction”)
3	#3 Search (“randomized controlled trial”[Title/Abstract]) OR (“randomized”[Title/Abstract]) OR (“randomly”[Title/Abstract])
4	#1 AND #2 AND #3

### 2.4 Literature screening and data extraction

**Figure 1:**
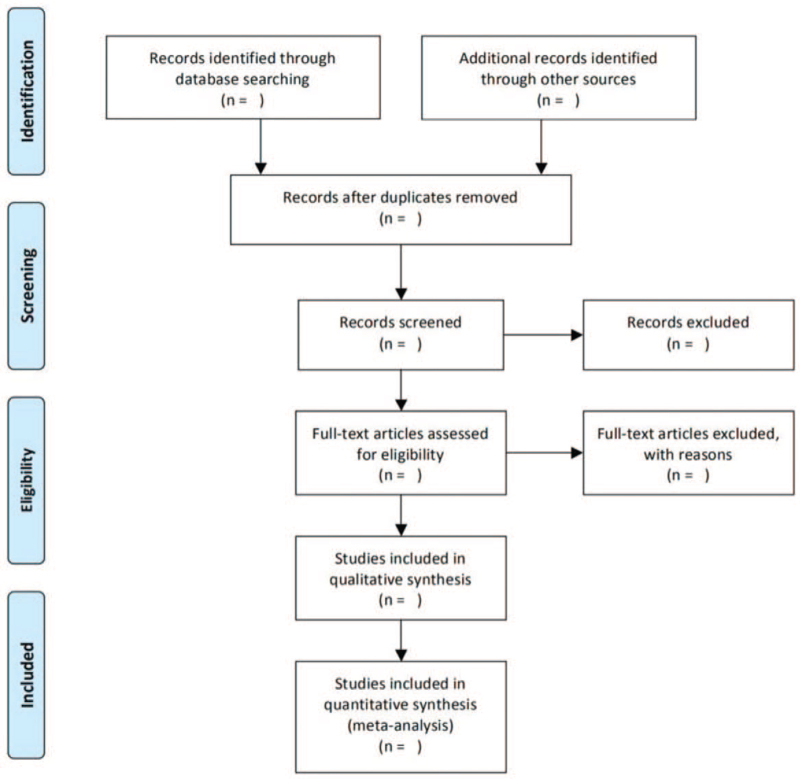
PRISMA flow diagram. PRISMA = preferred reporting items for systematic review and meta-analysis.

Two researchers independently conducted a study on the extraction of relevant information from a full-text reading of the literature. In addition, the preferred reporting items for systematic review and meta-analysis flow diagram of research selection was given in Figure 1.

The data extraction included the basic information of the literature (author, publication time, and publication journal), intervention methods (intervention measures, conventional therapeutic drugs, course of treatment, and dose), bias risk assessment (research design type, random method, distribution hiding, blind method, data integrity, outcome report, etc), relevant outcome indicators and adverse reactions. When the 2 parties disagree on the inclusion of the literature, the content will be discussed and evaluated by a third party, and the information of the final literature will be extracted.

### 2.5 Methodological quality evaluation

Included in the literature were evaluated according to the bias risk assessment tools of clinical RCT in the Cochrane manual. The evaluation items included randomized methods, allocation concealment, blind implementation of subjects and intervention providers, blind outcome evaluation, outcome data integrity, selective reporting, and other sources of bias. Each of the above items was judged by “low-risk,” “high-risk,” and “unclear.”

### 2.6 Data processing

The included literature research data were statistically analyzed by Rev Man5.3 software. The random-effect model was used for meta-analysis if the heterogeneity of the test results was large (*P* < .1, *I*^2^ > 50%); The fixed-effect model was used for meta-analysis if the test results were homogeneous (*P* > .1, *I*^2^ ≤ 50%). The counting data were analyzed by meta using the ratio as the combined statistic, and the mean difference as the statistic for meta-analysis. The standardized mean difference was used as the statistic for meta-analysis if different measurement methods or units were used, and sensitivity analysis and subgroup analysis were used if necessary. α = 0.05 was considered to be statistically significant. If meta-analysis was not possible, we only described the data.

### 2.7 Heterogeneity evaluation

The Revman5.3 software provided by the Cochrane website was used for analysis. The feasibility of meta-analysis of heterogeneity was assessed between trials. We will consider the existence of significant heterogeneity and conduct a subgroup analysis to investigate the potential causes of clinical or methodological heterogeneity if the *I*^2^ value was more than 50%.

### 2.8 Publication bias

The visual asymmetry on the funnel chart was used to determine whether there was a publication bias if there were at least 10 experimental studies.

### 2.9 Subgroup analysis

A subgroup analysis would be conducted according to different interventions, participants, gender, treatment duration, and drug dose, to explore the source of heterogeneity if there were at least 10 trials included.

### 2.10 Sensitivity analysis

Sensitivity analysis would be conducted according to sample size, missing data, and methodological quality to identify the quality.

### 2.11 GRADE evidence quality classification^[10]^

In view of the results of this systematic review, the GRADE evaluation tool was used to grade the evidence of the outcome indicators of each study. This evidence quality evaluation mainly considered the risk of quality bias, inconsistency of results, directness of evidence, the accuracy of evidence, and publication bias. The evidence quality was degraded when there are 1 or more of the above factors. The quality of evidence can be divided into 4 grades: high (-0), medium (-1), low (-2), and very low (-3). The recommended level was divided into strong and weak levels.

## 3 Conclusion

XKS is composed of *Salvia miltiorrhiza*, *Pueraria lobata*, *Panax notoginseng*, Hawthorn, and wood incense.^[11]^*Salvia miltiorrhiza* is the king medicine, which has the effect of promoting blood circulation and removing blood stasis, and relieving pain. At the same time, *Panax notoginseng* is the minister medicine, which has the function of promoting blood circulation and removing blood stasis, stopping blood stasis, and relieving pain. Besides, Hawthorn digestion and invigorating stomach, removing blood stasis, *Pueraria lobata* unimpeded qi, and dredge collaterals. The 3 medicines complement each other. The whole prescription has the effect of promoting blood circulation and removing blood stasis and relieving pain.^[12]^ It has been used in the treatment of CHD with qi stagnation and blood stasis syndrome for more than 30 years. It has been recognized as the first choice for CHD and the national protection variety of traditional Chinese medicine because of its definite clinical effect.^[13]^ XKS plays an important role in improving hemorheology, regulating blood lipids, protecting myocardial ischemia-reperfusion injury, improving vascular endothelial function, and inflammatory reaction.^[14]^ Moreover, it has a significant curative effect on CHD, hyperlipidemia, cardiac neurosis, bicardial disease, brain-heart syndrome, and so on.

This is a study to evaluate the clinical efficacy and safety of XKS in the treatment of AP of CHD. Generally speaking, this study proves that XKS combined with conventional western medicine can effectively reduce the frequency of AP, reduce the duration of AP, reduce the amount of nitroglycerin, improve the efficacy of electrocardiogram, the efficacy of AP, reduce triglycerides, total cholesterol, low-density lipoprotein cholesterol, and increase high-density lipoprotein cholesterol, lower whole blood viscosity, plasma viscosity, fibrinogen, C-reactive protein, endothelin-1, homocysteine, and improve nitric oxide. Clinical application of XKS in the treatment of AP of CHD is more instructive. However, at the same time, there are the following limitations: it may cause bias and affect the authenticity of clinical RCT if the literature included in the systematic review is not strictly randomized and blinded, and the allocation concealment is not mentioned; most studies do not specify the dosage and manufacturer in clinical application, which may increase clinical heterogeneity. Higher quality clinical RCT should be included to provide a higher evidence-based basis for clinical drug use. In the meanwhile, randomized methods should be defined in future clinical RCT, distribution concealment and blind methods should be implemented, detailed registration should be made for patients who lose follow-up, adverse reactions should be recorded in detail, and trial reports should be written in strict accordance with international CONSORT standard entries. It is necessary to add TCM evaluation indicators in TCM to improve the quality of clinical trials. In addition, it is committed to better clarifying the clinical efficacy of XKS and better improve the quality of life and prognosis of patients.

## Author contributions

**Conceptualization:** Maoxia Fan, Zhou Yu.

**Data curation:** Ying Tian, Yongcheng Liu.

**Formal analysis:** Maoxia Fan, Dong Guo, Jisen Zhao.

**Investigation:** Yiming Wang, Yongcheng Liu.

**Methodology:** Maoxia Fan, Yongcheng Liu, Yiming Wang.

**Project administration:** Jisen Zhao.

**Software:** Maoxia Fan, Jisen Zhao, Yiming Wang, Zhou Yu.

**Validation:** Jisen Zhao, Zhou Yu.

**Visualization:** Jisen Zhao.

**Writing – original draft:** Maoxia Fan, Dong Guo.

**Writing – review & editing:** Maoxia Fan, Dong Guo.
